# Sociodemographic determinants of intertrochanteric hip fractures in older adults: evidence from a six-year retrospective study in an Ecuadorian hospital

**DOI:** 10.3389/fsurg.2026.1732009

**Published:** 2026-04-13

**Authors:** Kevin S. Miranda, Lilian R. Calderón, Santiago Piedra, Miguel Ochoa-Andrade

**Affiliations:** Faculty of Medical Specialties, Universidad de Las Américas (UDLA), Quito, Ecuador

**Keywords:** intertrochanteric fracture, older adults, hip, epidemiology, Ecuador (country)

## Abstract

Intertrochanteric fractures are a major cause of morbidity, functional dependence, and mortality in older adults. This study aimed to describe the epidemiological profile and clinical outcomes of patients with intertrochanteric fractures treated at a referral center. A descriptive, observational, cross-sectional study was conducted that included 216 patients aged ≥65 years treated between 2019 and 2024. Most patients were women (65.7%) and belonged to the oldest-old subgroup (50%). The predominant mechanism of injury was a fall from standing height, and most fractures corresponded to unstable Tronzo patterns (67.1%). Cephalomedullary nailing was the primary surgical treatment (≈88%). The median preoperative hospital stay was 5 days, and total hospital stay was 8 days. In-hospital mortality was low (2.3%). In conclusion, intertrochanteric fractures most frequently affected older women after low-energy falls and were predominantly unstable patterns treated with intramedullary fixation, consistent with international evidence. Optimizing preoperative timelines and strengthening preventive strategies in older adults are necessary to improve outcomes.

## Introduction

Hip fractures constitute a major public health concern worldwide due to their association with increased mortality, functional decline, loss of independence, and substantial healthcare costs, particularly among older adults (1–3). As population aging accelerates globally, the burden of hip fractures is expected to increase, posing significant challenges to healthcare systems, especially in low- and middle-income countries.

Intertrochanteric hip fractures are extracapsular fractures occurring between the greater and lesser trochanters of the femur and represent a distinct clinical and biomechanical entity within the broader category of hip fractures. In contrast to intracapsular femoral neck fractures, intertrochanteric fractures are characterized by different fracture patterns, fixation strategies, complication profiles, and postoperative outcomes, which justify their separate epidemiological and clinical evaluation (4, 5).

These fractures predominantly affect older adults and are commonly associated with low-energy mechanisms, such as falls from standing height. Age-related physiological changes, comorbidity burden, functional impairment, and social vulnerability contribute to fracture risk and influence clinical outcomes. Previous studies have identified sex, advanced age, socioeconomic conditions, and living environment as relevant population-level characteristics associated with hip fracture occurrence; however, most available evidence originates from high-income countries, limiting its generalizability to Latin American healthcare settings (2, 6, 7).

In Ecuador, epidemiological data regarding intertrochanteric hip fractures and their sociodemographic context remain limited. Describing the population profile of affected patients is essential to understand local care demands, identify vulnerable groups, and inform future analytical and preventive strategies within the national healthcare system (2, 3).

Therefore, the aim of this study was to describe the sociodemographic and clinical characteristics of older adults with intertrochanteric hip fractures treated at a tertiary referral hospital in Ecuador over a six-year period. The term “determinants” is used in a descriptive epidemiological sense, referring to population-level sociodemographic characteristics rather than causal or predictive factors.

## Ethics approval

This study was conducted with authorization from the Human Research Ethics Committee of the Hospital General del Sur in Quito, part of the Ecuadorian Social Security Institute, with code CEISH-HGSQ-2025-014, dated May 26, 2025. The ethics committee waived specific informed consent. Information was extracted from patients' medical records by personnel not affiliated with the research team, in compliance with data protection laws, under the supervision of the Statistics and Planning Department of the San Francisco General Hospital in Quito. The data were anonymized before being accessed by the researchers.

## Materials and methods

### Study design and population

A retrospective, descriptive, single-center study was conducted at Hospital IESS San Francisco de Quito, a tertiary referral institution within the Ecuadorian Social Security Institute system. Medical records of patients aged 65 years and older admitted with a diagnosis of intertrochanteric hip fracture between January 2019 and December 2024 were reviewed. Fractures were identified using the ICD-10 code S72.1.

Inclusion criteria comprised patients aged ≥65 years with radiologically confirmed intertrochanteric hip fractures managed during the study period. Exclusion criteria included pathological fractures, periprosthetic fractures, incomplete medical records, and patients transferred to external institutions prior to definitive management.

Not all patients underwent surgical treatment at the study institution. Some patients were referred to other centers, managed conservatively, or died before surgery, which explains the discrepancy between the total cohort and the subset included in surgical and anesthetic analyses.

### Sociodemographic variables

Sociodemographic variables included age, sex, place of residence, educational level, occupational group, and type of social security beneficiary. Age was categorized according to World Health Organization criteria for older adults. Place of residence was classified by province and urban or rural setting. Educational level was grouped into no formal education, primary, secondary, and higher education. Occupational status was categorized using the International Standard Classification of Occupations (ISCO-08). Type of beneficiary was classified according to Ecuadorian Social Security Institute categories, reflecting employment status and access to healthcare services.

### Clinical variables

Fracture patterns were classified according to the Tronzo classification system (4). Comorbidity burden was assessed using the Charlson Comorbidity Index and categorized into low, moderate, and high-risk groups (6). Clinical variables included treatment modality, time to surgery, length of hospital stay, and in-hospital mortality.

### Statistical analysis

A descriptive statistical approach was employed. Categorical variables were summarized as frequencies and percentages, and continuous variables as medians with interquartile ranges. No inferential or multivariable analyses were performed, in accordance with the descriptive objectives of the study.

## Results

A total of 216 patients met the inclusion criteria. The cohort was predominantly female, with a high proportion of patients aged 85 years and older. Most fractures resulted from low-energy falls, and unstable fracture patterns were frequent.

Cephalomedullary nailing was the most commonly used fixation method among surgically treated patients. Median preoperative waiting time and total length of hospital stay were prolonged compared with reports from high-income countries.

**Figure 1:**
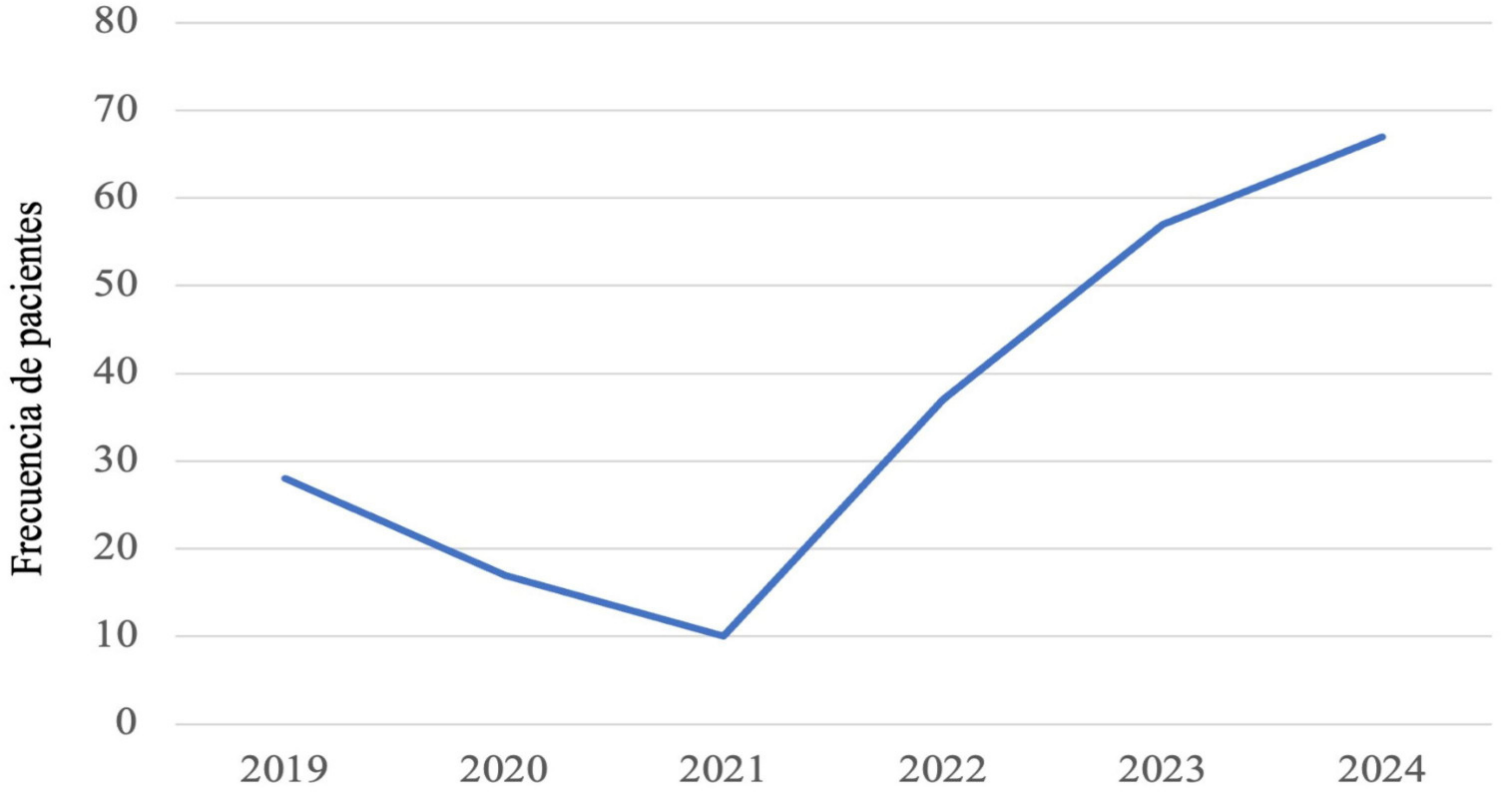
Frequency of patients with intertrochanteric fracture during the period 2019–2024.

**Figure 2:**
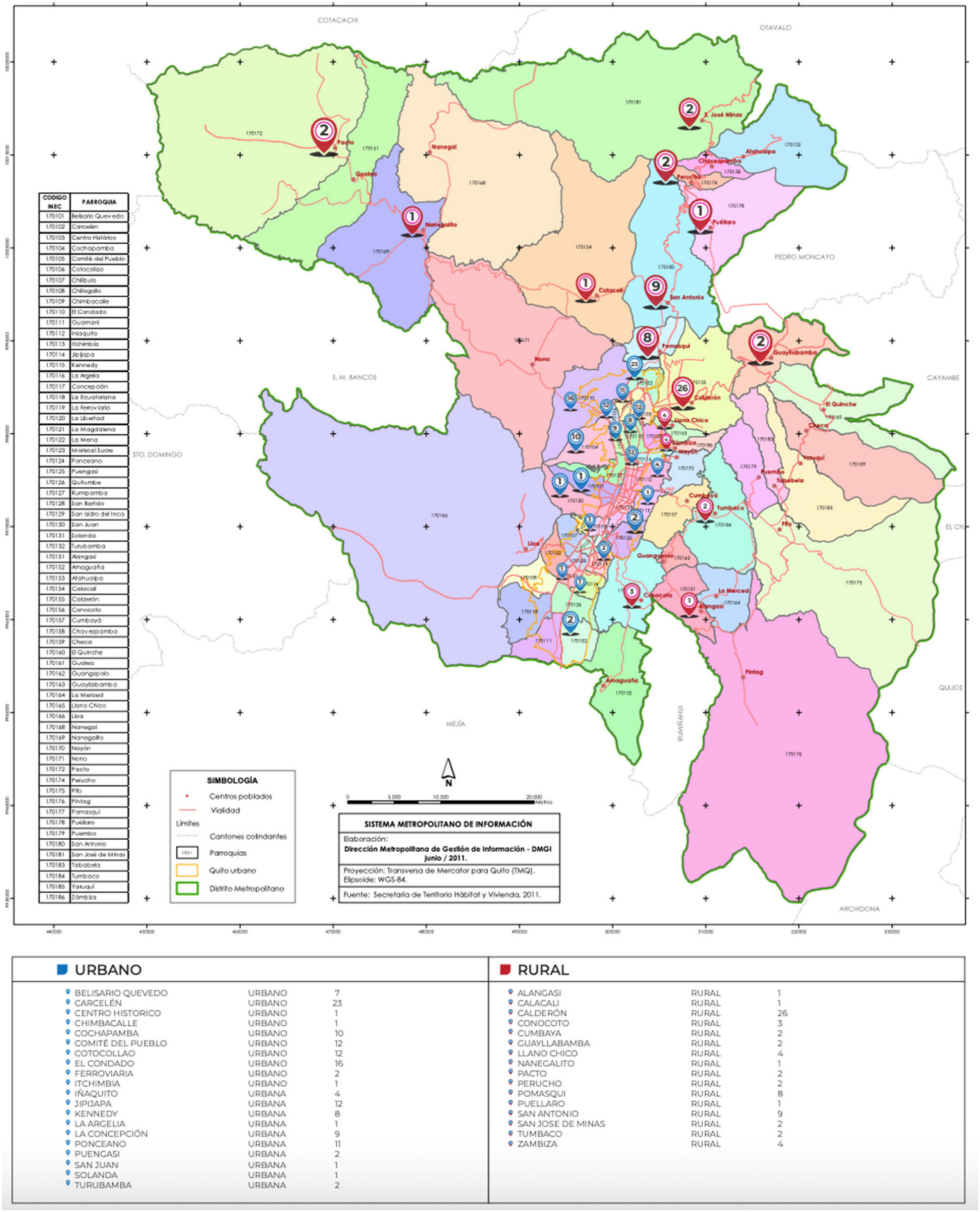
Geographical distribution of older adults treated for intertrochanteric fractures.

**Figure 3:**
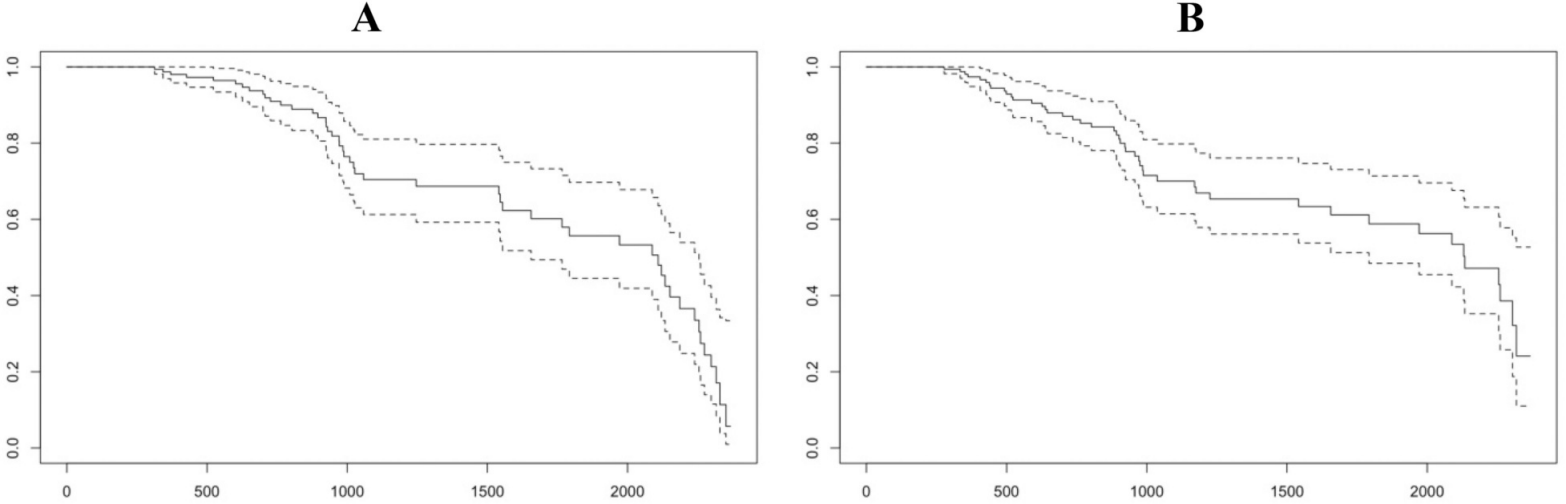
**(A)** Survival level in relation to the period from the date of surgery to the cut-off date (October 1, 2025), where x = days of follow-up and y = cumulative survival probability. **(B)** Association of mortality with the Charlson comorbidity index.

Detailed numerical data are presented in Tables 1–7 and Figures 1–3. Specifically, Tables 5–7 summarize treatment distribution, in-hospital mortality, and postoperative complications.

**Table 1:** Demographic data of older adults with intertrochanteric fractures.

Variable	Frecuency (*n*)	Percentage (%)
Sex (*n* = 216)
Male	74	34.26%
Female	142	65.74%
Age* (*n* = 216)		
Younger older adult (65–74 years)	33	15.28%
Intermediate older adult (75–84 years)	75	34.72%
Oldest old (≥85 years)	108	50.00%
Mean (DE) 83,68 (±8,23)		
Median (RIQ) 84,5 (78,5–90)		
Minimum–maximum age (65–100)		
Mode 83		
Province of residence (*n* = 216)		
Guayas	1	0.46%
Imbabura	3	1.39%
Pichincha	211	97.69%
Tungurahua	1	0.46%
Geographic area of residence (*n* = 216)		
Rural	140	64.81%
Urban	76	35.19%
Type of IESS + health affiliation^+^ (*n* = 216)		
Retiree	142	65.74%
Survivor's pension (Montepío)	32	14.81%
Rural retiree	17	7.87%
General insurance	7	3.24%
Voluntary	6	2.78%
Rural worker	5	2.31%
Rural head of household	3	1.39%
Retiree's spouse	3	1.39%
Spouse	1	0.46%
Occupational group^¶^ (*n* = 216)		
1	1	0.46%
2	43	19.91%
3	8	3.70%
4	9	4.17%
5	21	9.72%
6	18	8.33%
7	22	10.19%
8	8	3.70%
9	86	39.81%

* The age was classified by older adult age group according to the World Health Organization (WHO).

^+^ Ecuadorian Social Security Institute (IESS).

^¶^ The occupational group was classified according to the CIUO-08, with the following interpretation: (1) managers; (2) professionals; (3) technicians and associate professionals; (4) clerical support workers; (5) service and sales workers; (6) skilled agricultural, forestry, and fishery workers; (7) craft and related trades workers; (8) plant and machine operators, and assemblers; (9) elementary occupations and unskilled laborers.

**Table 2:** Clinical and kinematic characterization of the trauma.

Variable	Frecuency (*n*)	Percentage (%)
Mechanism of trauma (*n* = 216)		
Fall from standing height	214	99.07%
Traffic accident	2	0.93%
Laterality (*n* = 216)		
Left	110	50.93%
Rigth	106	49.07%
Intertrochanteric fracture classification* (*n* = 216)		
I	3	1.39%
II	33	15.28%
IIIA	53	24.54%
IIIB	92	42.59%
IV	14	6.48%
V	21	9.72%
Clinical classification (CCI)^+^ (*n* = 216)		
Low	143	66.20%
Moderate	57	26.39%
High	16	7.41%
Polypharmacy^¶^ (*n* = 216)		
No	155	71.76%
Yes	61	28.24%

* Fracture type was classified according to Tronzo.

^+^ The degree of short- and long-term mortality risk in patients with multiple comorbidities was assessed using the Charlson Comorbidity Index (CCI), calculated by summing points assigned to each comorbidity, with more severe conditions receiving higher scores: low (0–1 points), moderate (2–3 points), high (≥4 points).

^¶^ Polypharmacy was defined according to Masnoon et al.

**Table 3:** Surgical characterization of older adults with intertrochanteric fractures.

Variable	Frecuency (*n*)	Percentage (%)
Type of anesthesia (*n* = 163)		
Spinal	152	93.25%
General	10	6.13%
General due to failed spinal	1	0.61%
Type of surgery (*n* = 163)		
Open	33	20.25%
Closed	130	79.75%
Type of implant used (*n* = 163)		
TFN (Trochanteric Fixation Nail)	87	53.37%
PFN (Proximal Femoral Nail)	56	34.36%
DHS (Dynamic Hip Screw)	14	8.59%
DCS (Dynamic Condylar Screw)	3	1.84%
Anatomical Plate FP	1	0.61%
Partial Prosthesis (Austin-Moore)	1	0.61%
Partial Prosthesis (Thompson)	1	0.61%
Surgery duration in minutes (*n* = 163)		
Mean (DE) 93,34 (±39,77)		
Median (RIQ) 80 (70–110)		
Minimum–maximum on minutes (40–300)		
Mode 60		
Intraoperative blood loss in milliliters (mL) (*n* = 163)		
Mean (DE) 174.53 (±127.21)		
Median (RIQ) 150 (100–200)		
Blood loss in milliliters (mL), minimum–maximum (50–1,000)		
Mode 100		

**Table 4:** In-hospital management characteristics of older adults with intertrochanteric fractures.

Variable
Hospital stay prior to surgery (days) (*n* = 163)
Mean (DE) 5.66 (±3.9)
Median (RIQ) 5 (3–8)
Preoperative hospital stay (days) min–max (0–25)
Mode 3
Hospital stay after surgery (days) (*n* = 163)
Mean (DE) 4.7 (±7.33)
Median (RIQ) 3 (2–4)
Postoperative hospital stay (days) min–max (1–62)
Mode 2
Total hospital stay (days) (*n* = 163)
Mean (DE) 10.37 (±8.04)
Median (RIQ) 8 (6–12)
Total hospital stay (days) min–max (2–65)
Mode 5

**Table 5:** Distribution of older adults with intertrochanteric fractures treated in the hospital.

Variable	Frecuency (*n*)	Percentage (%)
Distribution of intertrochanteric fractures (*n* = 216)		
Surgical	162	75%
Referrals*	45	20.83%
Conservative	3	1.39%
Death prior to surgery	1	0.46%
Death after surgery	4	1.85%
Voluntary discharge	1	0.46%

* The causes of referral were as follows: 75.56% due to lack of specific material supply (*n* = 34); 8.89% due to malfunctioning equipment (*n* = 4); 8.89% due to inadequate treatment capacity (*n* = 4); 4.44% due to lack of equipment (*n* = 2); 2.22% due to absence of the service in the hospital service portfolio (*n* = 1).

**Table 6:** In-hospital mortality of older adults with intertrochanteric fractures.

Variable	Frecuency (*n*)	Percentage (%)
Patients (*n* = 216)		
Survivors	211	97.69%
Deaths prior to procedure	4	1.84%
Deaths after procedure	1	0.46%
Mortality rate	Calculation	Percentage (%)
Overall in-hospital mortality	(1 + 4)/216 × 100	2,31%
Preoperative mortality	4/216 × 100	1,85%
Postoperative mortality	1/216 × 100	0,46%

**Table 7:** Distribution of postoperative outcomes in older adults with intertrochanteric.

Complications	Frecuency (*n*)	Percentage (%)
Early^Δ^ (*n* = 163)		
No complications	90	54.87%
Respiratory	19	11.59%
Cardiovascular	14	8.54%
Hematologic	11	6.71%
Renal	8	4.88%
Neurologic	6	3.66%
Gastrointestinal	5	3.05%
Metabolic	4	2.44%
IAAS	3	1.83%
Hematologic and neurologic	2	1.22%
Cardiovascular and neurologic	1	0.61%
Cardiovascular and respiratory	1	0.61%
Hematologic and renal	1	0.61%
Respiratory and renal	1	0.61%
Late (*n* = 162)^β^		
No complications	152	93.8%
Infectious	5	3.1%
Medical	4	2.5%
Implant-related	2	1.2%

^Δ^ Immediate postoperative complications included respiratory system issues, such as exacerbation of COPD, acute-on-chronic bronchitis, and fat embolism, followed by cardiovascular complications, which included pulmonary thromboembolism and persistently elevated blood pressure. Hematologic complications comprised post-hemorrhagic anemia and electrolyte disturbances. Gastrointestinal complications included upper digestive bleeding and constipation; neurologic complications included altered consciousness and acute confusional state; metabolic complications included diabetes mellitus and electrolyte imbalance; renal complications included acute renal failure and exacerbation of chronic kidney disease; infectious complications were healthcare-associated infections (HAIs); and multisystem complications involved simultaneous impairment of multiple organs and systems.

^β^ Infectious complications were primarily superficial surgical site infections; non-infectious medical complications included heart failure, seizure episodes, anemia, and sacral ulcers; and implant-related complications involved migration of the cephalic screw.

## Discussion

This study provides a comprehensive descriptive profile of older adults with intertrochanteric hip fractures treated at a tertiary referral hospital in Ecuador. The predominance of advanced age and female sex is consistent with international epidemiological trends (1, 2, 7), reinforcing the global vulnerability of older women to fragility fractures.

The high proportion of fractures resulting from low-energy falls reflects age-related physiological changes, including impaired balance and reduced bone strength, commonly reported in similar populations (4, 6, 8). The predominance of unstable fracture patterns underscores the clinical complexity of intertrochanteric fractures in older adults and supports the widespread use of cephalomedullary fixation observed in this cohort (5, 8).

Beyond clinical characteristics, this study highlights the relevance of sociodemographic variables that are less frequently explored in the literature. Occupational classification, type of social security beneficiary, and place of residence may function as proxies for social vulnerability, healthcare access, and referral pathways within the Ecuadorian public health system. Their descriptive inclusion adds contextual value and provides a foundation for future analytical research.

The geographic distribution of patients likely reflects referral patterns, population density, and access to tertiary care rather than true regional incidence rates. This distinction is important to avoid overinterpretation of spatial analyses in single-center studies.

Although in-hospital mortality was low, hip fractures should be interpreted as sentinel events with potential long-term consequences. Survival data and comorbidity indices are therefore presented descriptively, without inferential interpretation, to avoid overstating associations in the absence of formal survival analyses.

Institution-specific challenges, such as prolonged preoperative waiting times and high referral rates, are particularly relevant in resource-limited healthcare systems and warrant further investigation (9, 10).

## Limitations

This study has several limitations. Its retrospective, single-center design limits external validity and generalizability. Retrospective data collection is susceptible to missing information, documentation bias, and unmeasured confounding.

The absence of a control group precludes estimation of relative risk and causal inference. Bone health indicators, such as bone mineral density measurements, prior osteoporosis diagnosis, or anti-osteoporotic treatment, were not available (3). Additionally, functional outcomes and post-discharge follow-up data were not assessed.

## Conclusions

This study provides a detailed descriptive characterization of older adults with intertrochanteric hip fractures treated in a tertiary Ecuadorian hospital. The findings highlight the interplay between advanced age, female sex, fracture instability, and social and healthcare access factors.

Given its descriptive design, the study does not allow causal or associative inference. However, it offers valuable epidemiological insight from an underrepresented region and establishes a foundation for future analytical studies aimed at evaluating determinants of outcomes and informing preventive strategies in similar healthcare settings.

## Data Availability

The original contributions presented in the study are included in the article/Supplementary Material, further inquiries can be directed to the corresponding author.
